# Tunable insulator-quantum Hall transition in a weakly interacting two-dimensional electron system

**DOI:** 10.1186/1556-276X-8-307

**Published:** 2013-07-03

**Authors:** Shun-Tsung Lo, Yi-Ting Wang, Sheng-Di Lin, Gottfried Strasser, Jonathan P Bird, Yang-Fang Chen, Chi-Te Liang

**Affiliations:** 1 Graduate Institute of Applied Physics, National Taiwan University, Taipei 106, Taiwan; 2 Department of Physics, National Taiwan University, Taipei 106, Taiwan; 3 Department of Electronics Engineering, National Chiao Tung University, Hsinchu 300, Taiwan; 4 Institute for Solid State Electronics and Center for Micro- and Nanostructures, Technische Universität Wien, Floragasse 7, 1040, Vienna, Austria; 5 Department of Electrical Engineering, University at Buffalo, The State University of New York, Buffalo, New York 14260-1920, USA

**Keywords:** Hall effect, Magnetoresistance, Electrons, Direct insulator-quantum hall transition

## Abstract

We have performed low-temperature measurements on a gated two-dimensional electron system in which electron–electron (e-e) interactions are insignificant. At low magnetic fields, disorder-driven movement of the crossing of longitudinal and Hall resistivities (*ρ*_xx_ and *ρ*_xy_) can be observed. Interestingly, by applying different gate voltages, we demonstrate that such a crossing at *ρ*_xx_ ~ *ρ*_xy_ can occur at a magnetic field higher, lower, or equal to the temperature-independent point in *ρ*_xx_ which corresponds to the direct insulator-quantum Hall transition. We explicitly show that *ρ*_xx_ ~ *ρ*_xy_ occurs at the inverse of the classical Drude mobility 1/*μ*_D_ rather than the crossing field corresponding to the insulator-quantum Hall transition. Moreover, we show that the background magnetoresistance can affect the transport properties of our device significantly. Thus, we suggest that great care must be taken when calculating the renormalized mobility caused by e-e interactions.

## Background

At low temperatures (*T*), disorder and electron–electron (e-e) interactions may govern the transport properties of a two-dimensional electron system (2DES) in which electrons are confined in a layer of the nanoscale, leading to the appearance of new regimes of transport behavior [1]. In the presence of sufficiently strong disorder, a 2DES may behave as an insulator in the sense that its longitudinal resistivity (*ρ*_xx_) decreases with increasing *T*[2]. It is useful to probe the intriguing features of this 2D insulating state by applying a magnetic field (*B*) perpendicular to the plane of a 2DES [2-4]. In particular, the direct transition from an insulator (I) to a high filling factor (*v* ≥ 3) quantum Hall (QH) state continues to attract a great deal of both experimental [5-13] and theoretical [14-16] interest. This is motivated by the relevance of this transition to the zero-field metal-insulator transition [17] and by the insight it provides on the evolution of extended states at low magnetic fields. It has already been shown that the nature of the background disorder, in coexistence with e-e interactions, may influence the zero-field metallic behavior [18] and the QH plateau-plateau transitions [19,20]. However, studies focused on the direct I-QH transitions in a 2DES with different kinds of disorder are still lacking. Previously, we have studied a 2DES containing self-assembled InAs quantum dots [11], providing a predominantly short-range character to the disorder. We observed multiple *T*-independent points in *ρ*_*xx*_(*B*), indicating a series of transitions between a low-field insulator and a QH state. The oscillatory amplitude of *ρ*_*xx*_(*B*) was well fitted by the Shubnikov-de Haas (SdH) theory [21-23], with amplitude given by

(1) $$\Delta \rho_{\mathrm{xx}}\left(B,T\right)=C\exp\left(-\pi /\mu_{q}B\right)D\left(B,T\right),$$

where *μ*_q_ represents the quantum mobility, *D*(*B*, *T*) = 2*π*^2^*k*_B_*m* * *T*/ℏ*eB* sinh (2*π*^2^*k*_B_*m* * *T*/ℏ*eB*), and *C* is a constant relevant to the value of *ρ*_xx_ at *B* = 0 T. The observation of the SdH oscillations suggests the possible existence of a Fermi-liquid metal. It should be pointed out that the SdH theory is derived by considering Landau quantization in the metallic regime without taking localization effects into account [24,25]. By observing the *T*-dependent Hall slope, however, the importance of e-e interactions in the metallic regime can be demonstrated [26]. In addition, as reported in [27], with a long-range scattering potential, SdH-type oscillations appear to span from the insulating to the QH-like regime when the e-e interaction correction is weak. Recently, the significance of percolation has been revealed both experimentally [28] and theoretically [29,30]. Therefore, to fully understand the direct I-QH transition, further studies on e-e interactions in the presence of background disorder are required.

At low *B*, quantum corrections resulting from weak localization (WL) and e-e interactions determine the temperature and magnetic field dependences of the conductivity, and both can lead to insulating behavior. The contribution of e-e interactions can be extracted after the suppression of WL at *B* >*B*_tr_, where the transport magnetic field (*B*_tr_) is given by $\frac{\hbar }{4\mathrm{eD\tau}}$ with reduced Planck's constant (ℏ), electron charge (*e*), diffusion constant (*D*), and transport relaxation time (*τ*). In systems with short-range potential fluctuations, the theory of e-e interactions is well established [31]. It is derived based on the interference of electron waves that follow different paths, one that is scattered off an impurity and another that is scattered by the potential oscillations (Friedel oscillation) created by all remaining electrons. The underlying physics is strongly related to the return probability of a scattered electron. In the diffusion regime (*k*_B_*Tτ*/ℏ < < 1 with Boltzmann constant *k*_B_), e-e interactions contribute only to the longitudinal conductivity (*σ*_xx_) without modifying the Hall conductivity (*σ*_xy_). On the other hand, in the ballistic regime (*k*_B_*Tτ*/ℏ > > 1), e-e interactions contribute both to *σ*_xx_ and *σ*_xy_, and effectively reduce to a renormalization of the transport mobility. However, the situation is different for long-range potential fluctuations, which are usually dominant in high-quality GaAs-based heterostructures in which the dopants are separated from the 2D electron gas by an undoped spacer. It is predicted that the interaction corrections can be suppressed at *B* = 0 but that they can eventually be restored at high magnetic fields *B* > 1/*μ*_D_ with enhanced return probability of scattered electrons, where *μ*_D_ represents the Drude mobility [32,33]. Therefore, it is of great interest to study the direct insulator-quantum Hall transition in a system with long-range scattering, under which the e-e interactions can be sufficiently weak at low magnetic fields.

Theoretically, for either kind of background disorder, no specific feature of interaction correction is predicted in the intermediate regime where *k*_B_*Tτ*/ℏ ≈ 1. Nevertheless, as generalized by Minkov et al. [34,35], electron–electron interactions can still be decomposed into two parts. One, with properties similar to that in the diffusion regime, is termed the diffusion component, whereas the other, sharing common features with that in the ballistic limit, is known as the ballistic component. Therefore, by considering the renormalized transport mobility *μ′* induced by the ballistic contribution and the diffusion correction $\delta \sigma_{\mathrm{xx}}^{d}$, *σ*_xx_ is expressed as

(2) σxx=neμ'1+μ'2B2+δσxxd,

(3) $$\sigma_{\mathrm{xy}}=\frac{\mathrm{ne\mu}{'}^{2}B}{1+\mu {'}^{2}B^{2}}.$$

It directly follows that the ballistic contribution $\delta \sigma_{\mathrm{xx}}^{b}$ is given by $\delta \sigma_{\mathrm{xx}}^{b}=\mathrm{ne}\left(\mu '-\mu_{D}\right),$ where *n* is the electron density and *μ*_D_ is the transport mobility derived in the Drude model. After performing matrix inversion with the components given in Equations 2 and 3, the magnetoresistance *ρ*_xx_(*B*) takes the parabolic form [36,37]

(4) ρxx≈1neμ'−1neμ'21−μ'2B2δσxxd

The Hall slope *R*_H_ (*ρ*_xy_/*B* with Hall resistivity *ρ*_xy_) now becomes *T*-dependent which is ascribed to the diffusion correction $\delta \sigma_{\mathrm{xx}}^{d}$[38]. As will be shown later, Equations 3, 4, and 5 will be used to estimate the e-e interactions in our system. Moreover, both diffusive and ballistic parts will be studied.

As suggested by Huckestein [16], at the direct I-QH transition that is characterized by the approximately *T*-independent point in *ρ*_xx_,

(5) $$\rho_{\mathrm{xx}},\rho_{\mathrm{xy}}$$

While Equation 5 holds true in some experiments [2], in others it has been found that *ρ*_xy_ can be significantly higher than *ρ*_xx_ near the direct I-QH transition [10,28]. On the other hand, *ρ*_xy_ can also be lower than *ρ*_xx_ near the direct I-QH transition in some systems [39]. Therefore, it is interesting to explore if it is possible to tune the direct I-QH transition within the *same* system so as to study the validity of Equation 5. In the original work of Huckestein [16], e-e interactions were not considered. Therefore, it is highly desirable to study a weakly disordered system in which e-e interactions are insignificant. In this paper, we investigate the direct I-QH transition in the presence of a long-range scattering potential, which is exploited as a means to suppress e-e interactions. We are able to tune the direct I-QH transition so that the corresponding field for which Equation 5 is satisfied can be higher or lower than, or even equal, to the crossing field that corresponds to the direct I-QH transition. Interestingly, we show that the inverse Drude mobility 1/*μ*_D_ is approximately equal to the field where *ρ*_xx_ crosses *ρ*_xy_, rather than the one responsible for the direct I-QH transition. We also show that the onset of strong localization occurs at a relatively higher field which does not correspond to 1/*μ*_D_.

## Methods

A gated modulation-doped AlGaAs/GaAs heterostructure (LM4640) is used in our study. The following layer sequence was grown on a semi-insulating GaAs substrate: 1 μm GaAs, 200 nm Al_0.33_Ga_0.67_As, 40 nm Si-doped Al_0.33_Ga_0.67_As with doping concentration in cubic centimeter, and finally a 10-nm GaAs cap layer. The sample was mesa etched into a standard Hall bar pattern, and a NiCr/Au gate was deposited on top of it by thermal evaporation. The length and width of the Hall bars are 640 and 80 μm, respectively. Four-terminal magnetotransport measurements were performed in a top-loading He^3^ system using standard ac phase-sensitive lock-in techniques over the temperature range 0.32 K ≤ *T* ≤16 K at three different gate voltages *V*_g_ = −0.125, −0.145, and −0.165 V.

## Results and discussion

**Figure 1:**
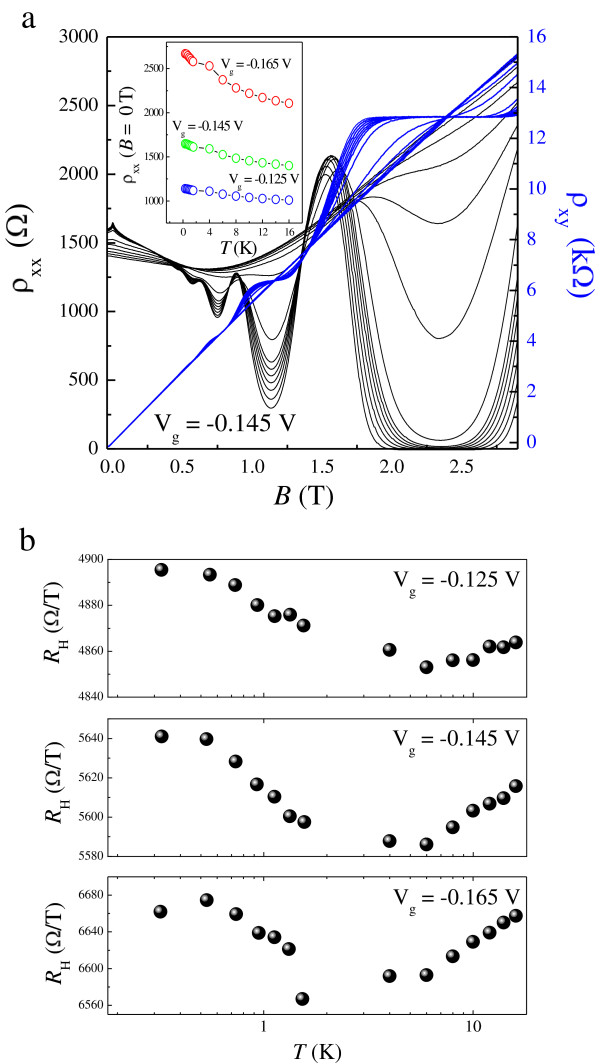
**Temperature dependence. (a)** Longitudinal and Hall resistivities (*ρ*_xx_ and *ρ*_xy_) as functions of magnetic field *B* at various temperatures *T* ranging from 0.3 to 16 K. The inset shows *ρ*_xx_(*B* = 0, *T*) at three applied gate voltages. **(b)** Hall slope *R*_H_ as a function of *T* at each *V*_g_ on a semi-logarithmic scale.

**Figure 2:**
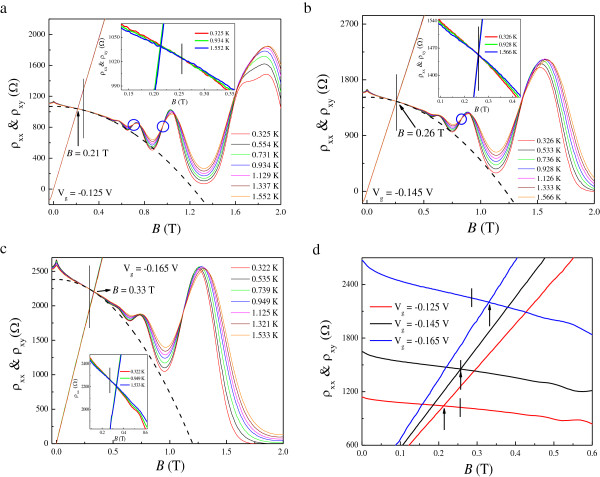
**Detailed results of *ρ***_**xx **_**and *ρ***_**xy **_**at low *T*.** The *B* dependences of *ρ*_xx_ and *ρ*_xy_ at various *T* ranging from 0.3 to 1.5 K for **(a)***V*_g_ = −0.125 V, **(b)***V*_g_ =−0.145 V, and **(c)***V*_g_ = −0.165 V. The insets are the zoom-ins of low-field *ρ*_xx_(*B*). The dashed lines are the fits to Equation 4 at the lowest *T*. For comparison, the results at the lowest *T* for each *V*_g_ are re-plotted in **(d)**. The *T*-independent points corresponding to the direct I-QH transition are indicated by vertical lines, and those for the crossings of *ρ*_xx_ and *ρ*_xy_ are denoted by arrows. Other *T*-independent points are indicated by circles.

**Figure 3:**
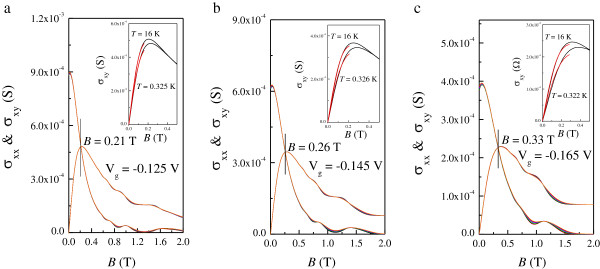
**Converted *σ***_**xx**_**(*B*) and *σ***_**xy**_**(*B*) at various *T *ranging from 0**.**3 to 1**.**5 K.** For **(a)***V*_g_ = −0.125 V, **(b)***V*_g_ = −0.145 V, and **(c)***V*_g_ = −0.165 V. The insets show *σ*_xy_(*B*) at *T* = 0.3 K and *T* = 16 K together with the fits to Equation 3 as indicated by the red lines. The vertical lines point out the crossings of *σ*_xx_ and *σ*_xy_.

**Figure 4:**
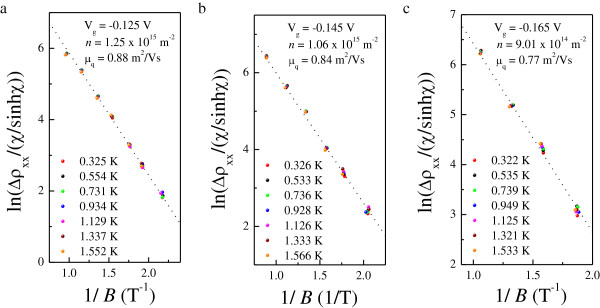
**ln (**Δ***ρ***_**xx**_**(*B*,*T*)/*D*(*B*,*T*)) as a function of 1**/***B*.** For **(a)***V*_g_ = −0.125 V, **(b)***V*_g_ = −0.145 V, and **(c)***V*_g_ = −0.165 V. The dotted lines are the fits to Equation 1.

Figure 1a shows *ρ*_xx_(*B*) and *ρ*_xy_(*B*) at various *T* for *V*_g_ = −0.145 V. It can be seen from the inset in Figure 1 that the 2DES behaves as an insulator over the whole temperature range at all applied gate voltages. The Hall slope *R*_H_ shows a weak *T* dependence below *T* = 4 K and is approximately constant at high *T*, which can be seen clearly in Figure 1b for each *V*_g_. For 1.84 T <*B* < 2.85 T, a well-developed *ν* = 2 QH state manifests itself in the quantized *ν* = 2 Hall plateau and the associated vanishing of *ρ*_xx_. In order to study the transition from an insulator to a QH state, detailed results of *ρ*_xx_ and *ρ*_xy_ at low *T* are shown in Figure 2a,b,c for each *V*_g_, and the converted *σ*_xx_ and *σ*_xy_ are presented in Figure 3. At *V*_g_ = −0.125 V, spin splitting is resolved as the effective disorder is decreased compared to that at *V*_g_ = −0.145 and −0.165 V. The reason for this is that the carrier density at *V*_g_ = −0.125 V is higher than those at *V*_g_ = −0.145 and −0.165 V. Following the suppression of weak localization, with its sharp negative magnetoresistance (NMR) at low magnetic fields, the 2DES undergoes a direct I-QH at *B* = 0.26, 0.26, and 0.29 T ≡ *B*_c_ for *V*_g_ = −0.125, −0.145, and −0.165 V, respectively, since there is no signature of *ν* = 2 or *ν* = 1 QH state near *B*_c_. We note that in all cases, *B*_c_ > 10 *B*_tr_. Therefore, it is believed that near the crossing field, weak localization effect is not significant in our system [37]. It is of fundamental interest to see in Figure 2d that the relative position of *B*_c_ with respect to that corresponding to the crossing of *ρ*_xx_ and *ρ*_xy_ is not necessarily equal. Following the transition, magneto-oscillations superimposed on the background of NMR are observed within the range 0.46 T ≤ *B* ≤ 1.03 T, 0.49 T ≤ *B* ≤ 1.12 T, and 0.53 T ≤ *B* ≤ 0.94 T for corresponding *V*_g_, the oscillating amplitudes of which are all well fitted by Equation 1. The results are shown in Figure 4a,b,c for three different *V*_g_. The good agreement with the SdH theory suggests that strong localization effects are not significant near *B*_c_. This is consistent with our previous results, performed on both a delta-doped quantum well with additional modulation doping [13] and a modulation-doped AlGaAs/GaAs heterostructure with a superlattice structure [27]. It follows that we can obtain the quantum mobility *μ*_q_ from the fits, which is expected to be an essential quantity regarding Landau quantization. The estimated *μ*_q_ are 0.88, 0.84, and 0.77 m^2^/Vs for *V*_g_ = −0.125, −0.145, and −0.165 V, respectively. Moreover, from the oscillating period in 1/*B*, the carrier density *n* is shown to be *T*-independent such that a slight decrease in *R*_H_ at low *T* does not result from the enhancement of carrier density *n*. Instead, these results can be ascribed to e-e interactions.

**Figure 5:**
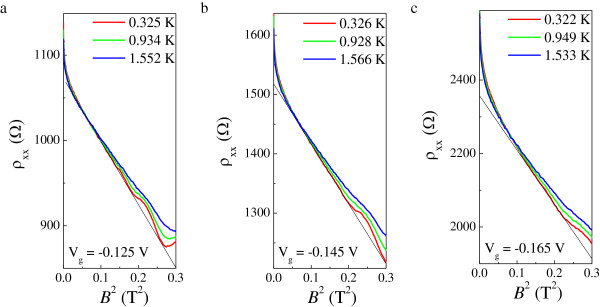
***ρ***_**xx **_**as a function of *B***^**2 **^**for *V***_**g **_**= −0.125 (a), −0.145 (b), and−0.165 (c) V.** The straight lines are provided as a guide to the eye to show the quadratic dependence on *B*.

**Figure 6:**
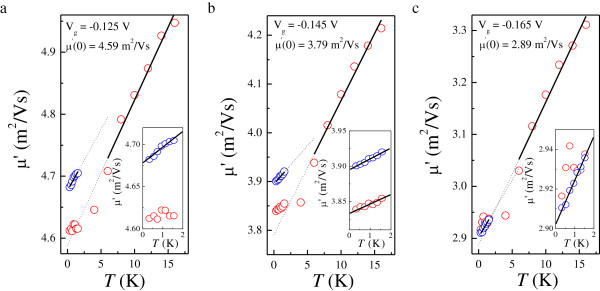
**Renormalized mobility *μ*′ as a function of *T *for *V***_**g **_**= −0.125 (a), −0.145 (b), and−0.165 (c) V.** The red and blue symbols denote the results obtained from the fits according to Equations 3 and 4, respectively. The insets are the zoom-ins of low-*T* results. The dotted lines represent the linear extrapolation of straight lines at *T* > 4 K.

At first glance, the *T*-dependent *R*_H_, together with the parabolic MR in *ρ*_xx_ (denoted by the dashed lines in Figure 2 for each *V*_g_), indicates that e-e interactions play an important role in our system. However, as will be shown later, the corrections provided by the diffusion and ballistic part of e-e interactions have opposite sign to each other, such that a cancelation of e-e interactions can be realized. Here we use two methods to analyze the contribution of e-e interactions. The first method is by fitting the measured *ρ*_xx_ to Equation 4, as shown by the blue symbols in Figure 5, from which we can obtain both $\delta \sigma_{\mathrm{xx}}^{b}$ and $\delta \sigma_{\mathrm{xx}}^{d}$. The value of $\delta \sigma_{\mathrm{xx}}^{d}$ is shown to be negative, as a result of the observed negative MR. We can see clearly from the dashed line in Figure 2 that the parabolic MR fits Equation 4 well at *B* >*B*_c_ but that it cannot be extended to the field where SdH oscillations occur. The obtained *μ′*, with an approximately linear dependence on *T* that is characteristic of the ballistic contribution of e-e interactions, is shown in Figure 6a,b,c for *V*_g_ = −0.125, −0.145, and −0.165 V, respectively. It should be mentioned that we cannot use this method to obtain *μ′* for *T* > 4 K since there is no apparent parabolic NMR, as shown in Figure 1a. The second method is based on the analysis of *σ*_xy_ using Equation 3, as shown in the inset to Figure 3 at the highest and lowest measured *T*. In this approach, *n* is determined from the SdH oscillations, from which the renormalized mobility can also be obtained at high *T* even without the parabolic negative MR induced by the diffusion correction. Here we limit the fitting intervals below 0.75 *B*_max_ to avoid the regime near *μ*_D_*B* ~ 1, where *B*_max_ denotes the field corresponding to the appearance of maximum *σ*_xy_ at the lowest *T*. The fitting results are plotted at each *V*_g_ as red symbols in Figure 6, allowing a comparison with those obtained by the first method. The figures show that *μ*′ is proportional to *T* when *T* > 4 K. There is a clear discrepancy between the values obtained from the different fits at a relatively lower magnitude of *V*_g_, which can be ascribed to the background MR (as will be discussed further below). Nevertheless, both cases indicate that the ballistic contribution, defined as $\delta \sigma_{\mathrm{xx}}^{b}=\mathrm{ne}\left(\mu '-\mu_{D}\right)$ with *μ*_D_ ≡ *μ*(*T* = 0*K*), has positive sign and therefore results in a partial cancelation of the diffusion correction. This is consistent with the prediction that the influence of e-e interactions is weakened in systems with long-range scattering potentials.

At high magnetic fields *B* > 1/*μ*_D_, semiclassical effects should affect the background resistance, resulting in either positive or negative MR [40,41]. Therefore, it is not possible to obtain reliable values for *μ*′ from the first method. Here we use the value of *μ*′(*T* = 0K), obtained by linearly extrapolating the high-*T* results from the second method to *T* = 0 K [27,34], to estimate *μ*_D_ and so as to allow a discussion on the role of the non-oscillatory background. As demonstrated in Figure 6, the estimated values of *μ*_D_ are 4.59, 3.79, and 2.89 m^2^/Vs for *V*_g_ = −0.125, −0.145, and −0.165 V, respectively, from which the corresponding ratios of *μ*_D_/*μ*_q_ (5.22, 4.51, and 3.75) are determined with *μ*_q_ obtained by analyzing the amplitudes of SdH oscillations as shown in Figure 3. Since *μ*_q_ counts all scattering events whereas *μ*_D_ is sensitive only to large-angle ones, we can deduce the predominant scattering mechanism in a 2DES from the value of *μ*_D_/*μ*_q_[42-44]. We can see from Figure 6 that both methods give the same results at low *T* for *V*_g_ = −0.165 V, implying that the influence of background MR is diminished as the amount of short-range scattering potential is increased. In what follows, we will focus on the issue about direct I-QH transitions.

Huckestein has suggested that the direct I-QH transition can be identified as a crossover from weak localization to the onset of Landau quantization, resulting in a strong reduction of the conductivity. The field *B* ~ 1/*μ* separates these two regions which are characterized by opposite *T* dependences and are characterized by *ρ*_xx_ ~ *ρ*_xy_. In his argument, *μ* is taken to be the transport mobility. Nevertheless, recent experimental results [11-13] demonstrate that different mobilities should be introduced to understand transport near a direct I-QH transition; the observed direct I-QH transition can be irrelevant to Landau quantization, while Landau quantization does not always cause the formation of QH states. Furthermore, it has already been demonstrated in various kinds of 2DES that the crossing point *ρ*_xx_ = *ρ*_xy_ can occur before or after the appearance of the *T*-independent point that corresponds to a direct I-QH transition_._ Moreover, the strongly *T*-dependent Hall slope induced by e-e interactions may affect the position of *ρ*_xx_ = *ρ*_xy_ at different *T*. As shown in Figure 2b for *V*_g_ = −0.145 V, the direct I-QH transition characterized by an approximately *T*-independent crossing point *B*_c_ in *ρ*_xx_ does occur at the field where *ρ*_xx_ ~ *ρ*_xy_ even though *ρ*_xy_ slightly depends on *T*. In addition, the inverse of the estimated Drude mobility 1/*μ*_D_ ~ 0.26 T is found to be close to *B*_c_. To this extent, Huckestein's model seems to be reasonable. However, we can see that there are no apparent oscillations in *ρ*_xx_ around *B*_c_ and that the onset of strong localization occurs at *B* > 1.37 T, as characterized by a well-quantized *ν* = 2 Hall plateau and vanishing *ρ*_xx_ with increasing *B*, more than five times larger than *B*_c_. In order to test the validity of the relation *ρ*_xx_ ~ *ρ*_xy_ at *B*_c_, different gate voltages were applied to vary the effective amount of disorder and carrier density in the 2DES. As shown in Figure 2a, by increasing *V*_g_ to −0.125 V, *ρ*_xx_ becomes smaller than *ρ*_xy_ at *B*_c_ ~ 0.26 T, while *ρ*_xx_ ~ *ρ*_xy_ at a smaller field of approximately 0.21 T, which is shown to be close to 1/*μ*_D_ ~ 0.22 T rather than *B*_c_. Moreover, by decreasing *V*_g_ to −0.165 V, *ρ*_xx_ ~ *ρ*_xy_ appears at *B* ~ 0.33 T which is larger than *B*_c_ ~ 0.29 T, as shown in Figure 2c. The inverse Drude mobility 1/*μ*_D_ ~ 0.35 is also found to be close to the field where *ρ*_xx_ ~ *ρ*_xy_ under this gate voltage. In all three cases, the crossings of *σ*_xx_ and *σ*_xy_ coincide with those of *ρ*_xx_ and *ρ*_xy_, as shown in Figure 2 for each *V*_g_. Therefore, our studies suggest that the field where *ρ*_xx_ ~ *ρ*_xy_ is governed by 1/*μ*_D_ and does not always correspond to that responsible for a direct I-QH transition as the influence of e-e interactions is not significant. As a result, *ρ*_xx_ ~ *ρ*_xy_ can occur on both sides of *B*_c_ as seen clearly in Figure 2d.

Interestingly, in the crossover from SdH oscillations to the QH state, we observe additional *T*-independent points, labeled by circles in Figure 2 for each *V*_g_, other than the one corresponding to the onset of strong localization. As shown in Figure 2a for *V*_g_ = −0.125 V, the resistivity peaks at around *B* = 0.73 and 1.03 T appear to move with increasing *T*, a feature of the scaling behavior [7] of standard QH theory around the crossing points *B* = 0.70 and 0.96 T, respectively. Therefore, survival of the SdH theory for 0.46 T ≤ *B* ≤ 1.03 T reveals that semiclassical metallic transport may coexist with quantum localization. The superimposed background MR may be the reason for this coexistence, which is demonstrated by the upturned deviation from the parabolic dependence as shown in Figure 2a [45]. Therefore, it is reasonable to attribute the overestimated *μ′* shown by the blue symbols in Figure 5a to the influence of the background MR. Similar behavior can also be found for *V*_g_ = −0.145 V even though spin splitting is unresolved, indicating that the contribution of background MR mostly comes from semiclassical effects. However, such a crossing point cannot be observed for *V*_g_ = −0.165 V since there is no clear separation between extended and localized states with strong disorder. Only a single *T*-independent point corresponding to the onset of strong localization occurs at *B* = 1.12 T.

**Figure 7:**
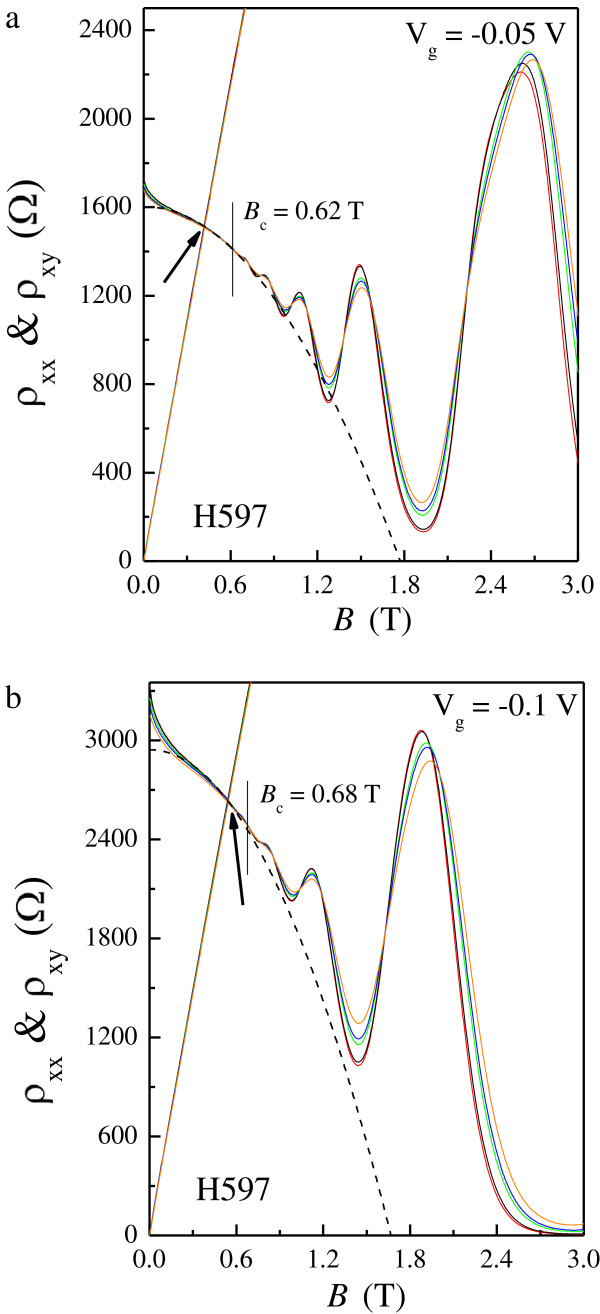
***ρ***_**xx **_**and *ρ***_**xy **_**as functions of *B *at various *T *ranging from 0. 3 to 2 K.** For **(a)***V*_g_ = −0.05 V and **(b)***V*_g_ = −0.1 V.

In order to check the validity of our present results, further experiments were performed on a device (H597) with nominally *T*-independent Hall slope at different applied gate voltages [27]. As shown in Figure 7a for *V*_g_ = −0.05 V, weakly insulating behavior occurs as *B* < 0.62 T ≡ *B*_c_, which corresponds to the direct I-QH transition since there is no evidence of the *ν* = 1 or *ν* = 2 QH state near *B*_c_. The crossing of *ρ*_xx_ and *ρ*_xy_ is found to occur at *B* ~ 0.5 T which is smaller than *B*_c_. As we decrease *V*_g_ to −0.1 V, thereby increasing the effective amount of disorder in the 2DES, the relative positions between these two fields remain the same as shown in Figure 7b. Nevertheless, it can be observed that *ρ*_xy_ tends to move closer to *ρ*_xx_ with decreasing *V*_g_. This may be quantified by defining the ratio *ρ*_xy_/*ρ*_xx_ at *B*_c_, whose value is 1.57 and 1.31 for *V*_g_ = −0.05 and −0.1 V, respectively.

**Figure 8:**
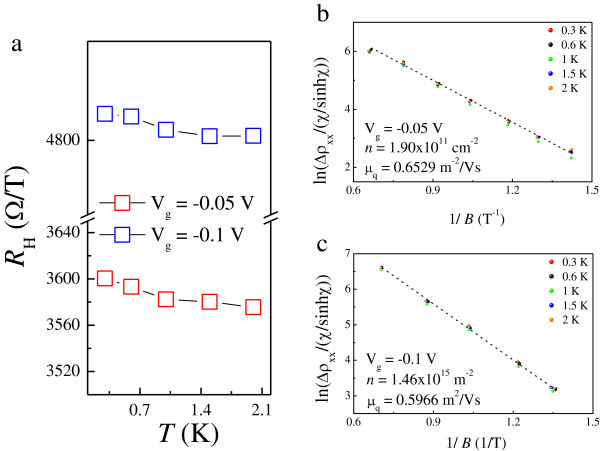
***R***_**H **_**and ln(**Δ***ρ***_**xx**_**(*B*,*T*)/*D*(*B*,*T*)). (a)***R*_H_ as a function of *T* for both gate voltages. ln(Δ*ρ*_xx_(*B*, *T*)/*D*(*B*, *T*)) as a function of 1/*B* is shown in **(b)** and **(c)** for *V*_g_ = −0.05 and −0.1 V, respectively. The dotted lines are the fits to Equation 1.

**Figure 9:**
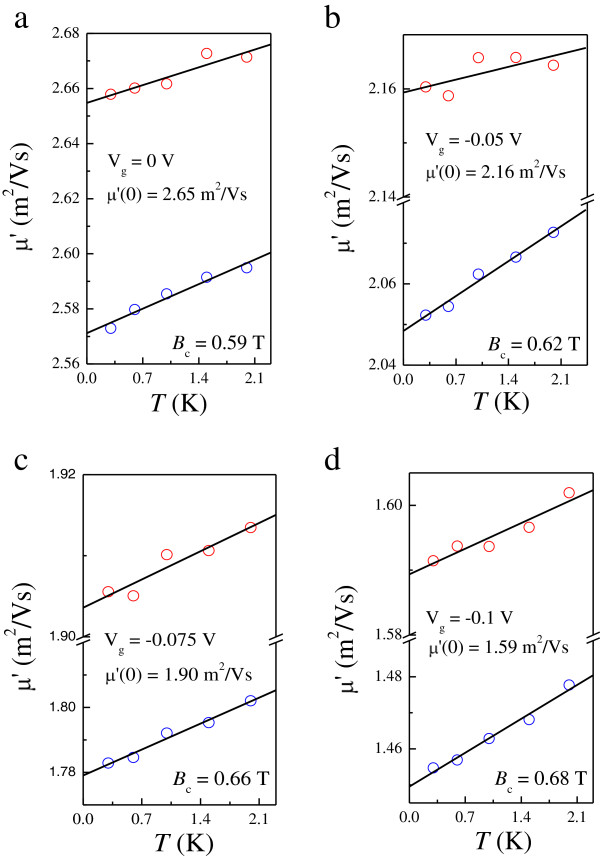
***μ′ *as a function of *T. *** For **(a) ***V*_g_ = 0 V, **(b)***V*_g_ = −0.05 V, **(c)***V*_g_ = −0.075 V, and **(d)***V*_g_ = −0.1 V. The symbols are the same as those used in Figure 6.

The interaction-induced parabolic NMR can be observed at both gate voltages. This result, together with the negligible *T* dependence of the Hall slope as shown in Figure 8a, implies that the ballistic part of the e-e interactions dominates as mentioned above. Therefore, by analyzing the observed parabolic NMR and corresponding Hall conductivity with Equations 4 and 3, respectively, we can obtain the renormalized transport mobilities *μ′* at each measured *T*. Again, the estimated *μ′* obtained by different methods as shown using different symbols in Figure 9 do not coincide with each other. It has already been demonstrated that the background MR can validate the SdH theory at *B* > 1/*μ*_q_ for *V*_g_ = −0.075 V in [27]. However, as shown in Figure 9c for *V*_g_ = −0.1 V, 1/*μ*_q_ ~ 1.67 T is found to be close to the crossing point in *ρ*_xx_ at *B* ~ 1.63 T, which corresponds to the *ν* = 4 to *ν* = 2 QH plateau-plateau transition. Therefore, it is reasonable to attribute the discrepancy of *μ′* obtained by different methods to the background MR. However, we can see that the value of *μ′* is underestimated by using the first method, which is different from that in sample LM4640 with the overestimated result. Our experimental results in conjunction with existing reports [37,45-48] suggest that a detailed treatment of the background MR is required. Moreover, the role of spin splitting does not seem to be significant in our system [49-51].

The inverse Drude mobilities 1/*μ*_D_ estimated by the same procedures are 0.38, 0.46, 0.53, and 0.63 T for *V*_g_ = 0, −0.05, −0.075, and −0.1 V, respectively. We can see clearly that 1/*μ*_D_ deviates from the crossing of *ρ*_xx_ and *ρ*_xy_ (0.35, 0.43, 0.47, and 0.54 T for the corresponding *V*_g_) as the applied gate voltage is decreased. The enhancement of background disorder with decreasing *V*_g_ may be the reason for such a discrepancy which can be deduced from the ratio *μ*_D_/*μ*_q_ (4.27, 3.32, 2.92, and 2.65 for the corresponding *V*_g_). The underlying physics is that the interference-induced e-e interactions are regained as a sufficient amount of short-range scattering potential is introduced, which leads to increased electron backscattering. Moreover, the parabolic NMR extending well below 1/*μ*_D_, as shown in Figure 7, provides another evidence for the recovery of e-e interactions since in a 2DES dominated by a long-range scattering potential, it occurs only as *B* > 1/*μ*_D_. We hope that our results will stimulate further investigations to fully understand the evolution of extended states near *μ*_D_*B* = 1 in a disordered 2DES both experimentally and theoretically.

## Conclusion

In conclusion, we have studied magnetotransport in gated two-dimensional electron systems. By varying the effective amount of disorder and the carrier density through different applied gate voltages, we observe that the crossing of *ρ*_xx_ and *ρ*_xy_ is governed by the inverse of the Drude mobility 1/*μ*_D_ and can occur for *B* >*B*_c_, *B* <*B*_c_, and *B* ~ *B*_c_ where *B*_c_ corresponds to the direct I-QH transition as the influence of e-e interactions is not significant. However, such a criterion breaks down when a sufficient amount of disorder is introduced, which leads to the recovery of interference-induced e-e interactions. Moreover, our results demonstrate that the magneto-oscillations following the semiclassical SdH theory can coexist with quantum localization as a result of the background MR, and the onset of strong localization occurs at a much higher field than either *B*_c_ or 1/*μ*_D_. Therefore, in order to obtain a thorough understanding of the ground state of a weakly interacting 2DES, it is essential to eliminate the influence of e-e interactions as much as possible.

## Competing interests

The authors declare that they have no competing interests.

## Authors’ contributions

STL and YTW performed the experiments. GS and SDL prepared the devices. YFC and CTL coordinated the project. STL, JPB, and CTL drafted the paper. All the authors read and approved the final version of the manuscript.
